# Viscosity-Regulated Control of RNA Microstructure Fabrication

**DOI:** 10.3390/polym13030454

**Published:** 2021-01-31

**Authors:** Sunghyun Moon, Hyejin Kim, Dajeong Kim, Jong Bum Lee

**Affiliations:** Department of Chemical Engineering, University of Seoul, Seoul 02504, Korea; shmoonuos@gmail.com (S.M.); ajin2010@uos.ac.kr (H.K.); djkim@uos.ac.kr (D.K.)

**Keywords:** RNA microstructures, viscosity, reaction control, RNA self-assemblies, crystallization

## Abstract

The development of RNA self-assemblies offers a powerful platform for a wide range of biomedical applications. The fabrication process has become more elaborate in order to achieve functional structures with maximized potential. As a facile means to control the structure, here, we report a new approach to manipulate the polymerization rate and subsequent self-assembly process through regulation of the reaction viscosity. As the RNA polymerization rate has a dependence on solution viscosity, the resulting assembly, crystallization, and overall sizes of the product could be manipulated. The simple and precise control of RNA polymerization and self-assembly by reaction viscosity will provide a way to widen the utility of RNA-based materials.

## 1. Introduction

RNA nanotechnology has advanced extensively for its potential biomedical applications by leveraging a wide range of biological functions of RNA. Additionally, the versatility of RNA for bottom-up self-assembly to achieve the defined size and structures gives a further advantage to fully exploit the therapeutic potential of RNA [1,2,3,4]. In particular, rolling circle transcription (RCT)-based enzymatic RNA self-assembly has gained much attention as one of the promising approaches to enhancing the loading capacity and functionality of therapeutic RNA, including messenger RNAs, small interfering RNAs, and microRNAs, both in vitro and in vivo [5,6,7,8].

RCT is a sophisticated process that generates RNA strands and pyrophosphate (PPi^4−^) as side products. Meanwhile, the Mg^2+^ cation in the reaction added as a co-factor for the enzyme forms a complex with pyrophosphate, resulting in magnesium pyrophosphate (Mg_2_PPi; Mg_2_P_2_O_7_) [9]. The inorganic pyrophosphate, Mg_2_PPi, is a biocompatible compound and a naturally occurring biological molecule [10]. Mg_2_PPi precipitate out from the solution and form crystalline structures with the adsorption of produced RNA and finally assemble into a unique sponge-like structure [11,12,13]. Specifically, RNA acts as a nucleation promoter at the initial stage and an inhibitor of crystal growth at the later stage through the interaction of RNA with the crystal surface. Overall, the major factors in RNA structure fabrication are Mg_2_PPi crystallization and RNA hybridization. 

In a previous study, RNAi microsponges were fabricated by RCT to deliver siRNA [14]. However, the large sizes of the microsponges hampered the cellular uptake. Thus, there have been several attempts to regulate the size of RNA-based structures by manipulating reaction components to potentiate the function of RNA and expand the utility of the material. For example, the size of the RNA structure was reduced by increasing polymerase concentration [5,15]. Other reaction components, such as circular DNA (template) and ribonucleotide triphosphate (monomer) were also manipulated to control the size of RNA structures [16,17]. Although the size regulation resulted in enhanced delivery efficiency, high costs due to expensive enzymes and unpredictable size regulation trends still need to be improved. 

**Figure 1:**
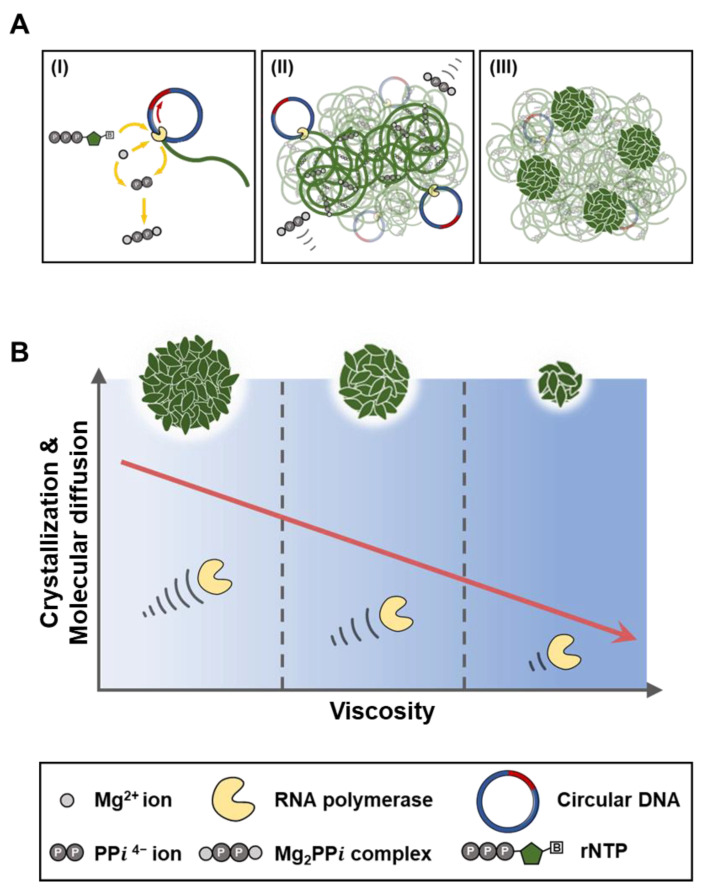
Schematic illustration of (**A**) RNA microstructures fabrication and (**B**) regulation of synthesis through viscosity manipulation.

In this context, we controlled the enzymatic RNA polymerization by regulating viscosity to manipulate external reaction conditions and studied how viscosity affects the RNA microstructures fabrication by altering the enzyme activities and molecular interactions between reaction components (Figure 1). Furthermore, the size control was demonstrated by regulating a single factor on a scale of 1.3 μm to 300 nm. The simple process of manipulation and predictable size-reduction trends offers multiple design choices to fit the purpose of biomedical application such as direct nucleic acid delivery to the tumor site (<500 nm) [18,19], immune cell-targeted nucleic acid delivery for immunotherapy (>500 nm) [20], lung-targeted nucleic acid delivery (>1 μm) [21], etc. Moreover, a variety of post-functionalities can be further applied to the surface of size-tuned RNA microstructures to provide tools such as targeting ability, stealthy property, or other functionalities [22,23]. Finally, it will provide the chance to understand the interaction between various sizes of RNA microstructures and living systems, which should be further explored and advanced to improve the nucleic acid-based therapeutics.

## 2. Materials and Methods

### 2.1. Materials

All oligonucleotides were purchased from Integrated DNA Technologies (IDT, Coralville, IA, USA). T7 RNA polymerase, RNA polymerase reaction buffer (40 mM Tris, 6 mM magnesium chloride, 2 mM spermidine, 1 mM dithiothreitol; pH 7.9), and ribonucleotide triphosphates (rNTPs) were purchased from New England BioLabs (NEB, Ipswich, MA, USA). Eighty percent glycerol solution was purchased from Intron Biotechnology (Seongnam, South Korea). Sodium pyrophosphate and magnesium chloride solutions were purchased from Sigma Aldrich (Saint Louis, MI, USA).

### 2.2. Calculation of Viscosity Containing Different Glycerol Contents

The viscosity of the solution with different glycerol contents was calculated by a modified equation derived from the Stokes-Einstein equation (D = kT/6πηR, where D is the diffusion coefficient, T is the temperature, k represents the Boltzmann’s constant, η is the viscosity of diffusion medium) [24]. The 100 nm polystyrene latex standard beads in the different glycerol contents were measured three times with 20 runs by dynamic light scattering (DLS) using Nano-ZS90 (Malvern, Worcestershire, UK ), operated at 25 °C, measurement angle at 90°. When the diffusion medium, temperature, and radius of a particle are held constant, the equation can be simplified into

η_1_R_1_ = η_2_R_2_ (1)

where η_1_ is the viscosity value introduced into software, R_1_ is the measured value of particle size, η_2_ is the actual solution viscosity, R_2_ is the known radius of the beads. The measured particle sizes were introduced into the equation, and the viscosity of the solution was calculated.

### 2.3. Fabrication of RNA Microstructures

Sense and anti-sense circular DNAs were synthesized from linear and primer DNA, as previously reported [25] (Table 1). Then, 10 μM of sense and anti-sense circular DNA, 1 mM of rNTP, 50 U of T7 RNA polymerase, RNA polymerase reaction buffer, and nuclease-free water were mixed. For the synthesis of viscosity-mediated size-controlled RNA microstructures, 80% glycerol solution was added to the reaction solution to make specific viscosity instead of nuclease-free water. The mixture was incubated at 37 °C for 20 h. Then, the product was washed 3 times by centrifuging at 12,000× *g* for 10 min. To synthesize larger particles, the initial circular DNA concentration was decreased from 10 μM to 3 μM, and the final rNTP concentration was increased from 1 mM to 2 mM. 

### 2.4. Formation of Magnesium Pyrophosphate Crystals

Sodium pyrophosphate was solubilized at a concentration of 20 mM into nuclease-free water. Magnesium chloride was diluted to 16 mM with nuclease-free water. Next, sodium pyrophosphate and magnesium chloride solution were simply mixed by 1:1 volume ratio, and 80% glycerol solution was added at the desired concentration (5%, 30%, or 50% (*v*/*v*)). Then, the mixture was incubated for 30 min at room temperature.

### 2.5. Analysis of RNA Polymerization Rate

For the assessment of RNA polymerization reaction rate at different time points, each reaction solution was incubated at 70 °C to inactivate RNA polymerase and diluted to achieve the same viscosity for all samples. After dilution, 50 mM ethylenediaminetetraacetic acid (EDTA, Sigma Aldrich, Saint Louis, MI, USA) was added to the final concentration of 10 mM to destroy the magnesium pyrophosphate crystalline structure. For the agarose gel electrophoresis analysis, each sample was loaded with 4X Gel loading dye (NEB, Ipswich, MA, USA) and 1X GelRed (Biotium, Fremont, CA, USA) and loaded to 1% agarose gel in Tris-acetate-EDTA buffer (Koma Biotechnology, Seoul, South Korea) and carried out at 100 V for 60 min and analyzed with GelDoc EZ imager (Bio-rad, Hercules, CA, USA). For the polyacrylamide gel electrophoresis (PAGE) analysis, 10% polyacrylamide gel was prepared and carried out at 100 V for 60 min in 1X tris-borate-EDTA buffer (Lonza, Alpharetta, GA, USA). Then, the gels were stained with 1X GelRed in 1X tris-borated-EDTA buffer for 20 min and analyzed as mentioned above. To obtain RNA yields from the gel image, the signal intensity for each lane was analyzed and compared to that of the single-strand RNA ladder (NEB, Ipswich, MA, USA). The PAGE analysis was performed three times, and the RNA yields were averaged.

### 2.6. Characterization of Fabricated RNA Microstructures

Scanning electron microscopy (SEM) images were captured using SNE-3000M (SEC Co., Ltd., Suwon, South Korea), operating at an accelerating voltage of 15 kV. Samples were prepared by dropping a diluted particle solution into a silicon wafer. The number averages of RNA microstructures fabricated at each viscosity was measured by DLS measurement, operated at 25 °C, measurement angle at 90°, three times with 20 runs. 

## 3. Results and Discussion 

**Figure 2:**
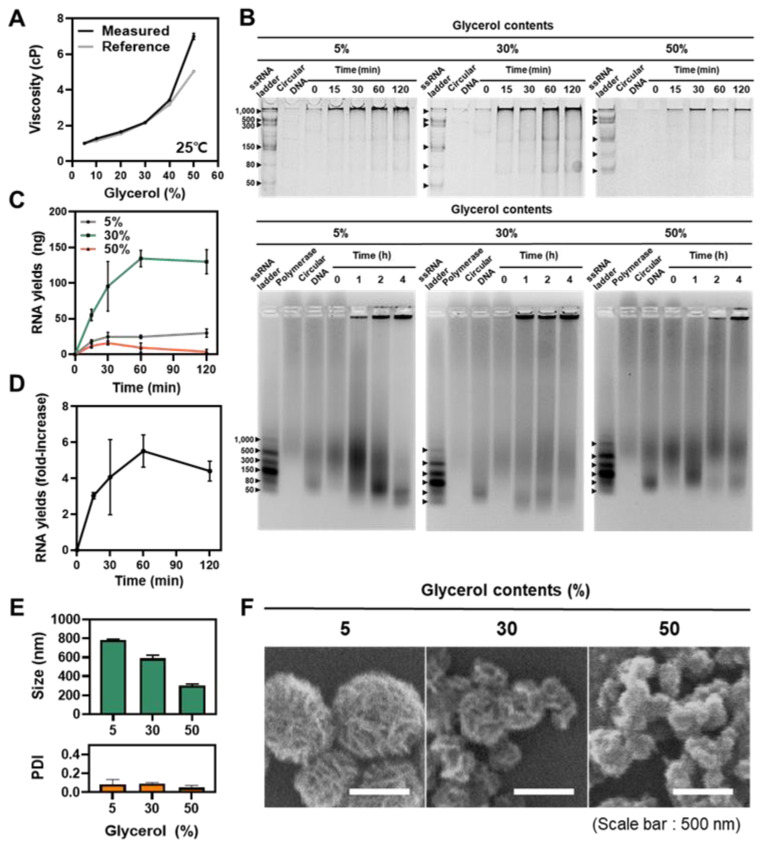
Different RNA polymerization rate and final products induced by manipulation of solution viscosity (template DNA to monomer = 10 × 10 − 3). (**A**) Manipulation of solution viscosity by increasing glycerol contents (n = 3). (**B**) One percent polyacrylamide gel electrophoresis and 10% agarose gel electrophoresis are exerted to analyze the rate of early-stage RNA polymerization rate at each solution viscosity. (**C**) RNA yields at each time point were acquired from electrophoresis data (n = 3). (**D**) Fold increases of RNA production at 30% glycerol contents compared to 5% glycerol contents. (**E**) The sizes, polydispersity index (PDI) and (**F**) SEM images of the final products with different glycerol contents after 20 h reaction (scale bar: 500 nm).

For the manipulation of solution viscosity, we chose glycerol as an additive. Glycerol is a viscous liquid due to the presence of three hydroxyl groups in the molecular structure, which is usually added in the enzymatic reaction medium to confer long-term stability of the enzyme. As the glycerol contents in the reaction increase, the viscosity of the reaction medium increases (Figure 2A). In addition, the experimental viscosity values obtained by using a defined reference material in DLS were consistent with a theoretical viscosity [24]. 

Next, we investigated the effect of manipulating the solution viscosity in the RNA polymerization reaction (Figure 2B–D). To analyze the polymerization rate, we quantified total RNA yields depending on the reaction time. As shown in Figure 2B, the yields were increased as glycerol contents increased from 5% to 30%. Even at 1 h after the reaction, RNA yields were increased by about 4.5-fold with the increases in viscosity, implying that a viscous environment is beneficial for efficient polymerization (Figure 2D). However, RNA polymerization was inhibited with a steep increase in viscosity at 50% glycerol condition, suggesting that the enzyme activity was affected severely, which is consistent with previous studies of the reduced protein activity in a highly viscous solution [26,27]. Of note, the RNA products synthesized in all viscosity conditions were already longer than 1000 bp at only 15 min (Figure 2B), indicating the RCT reaction still allows for a long RNA synthesis even in an unfavorable condition. Further, with 10% agarose gel electrophoresis, which has larger pore sizes than 1% polyacrylamides, the differences in RNA polymerization rate are more clearly compared. At 5%, 30% glycerol contents, the polymerized RNA, which is much longer than the 1000 bp needed to be caught in the well, is synthesized after 60 min. In contrast, this could be observed at 120 min at 50% glycerol contents. 

To evaluate the effect of an altered reaction rate on the final products, the size of the RNA microstructures was assessed. As glycerol contents increase from 5% to 50%, the size was reduced gradually from 782.5 (±7.85) to 301.6 (±15.29) nm (Figure 2E,F). For the 5–30% region, the reduced size of RNA microstructures is the result of inhibited crystallization caused by increased RNA products, as nucleic acid is known to act as an inhibitor of crystalline growth by adhering to the interfaces of a crystal structure [12]. However, the viscosity and limited molecular diffusion inhibit inorganic crystallization directly in 50% condition as no increase in RNA was observed. 

**Figure 3:**
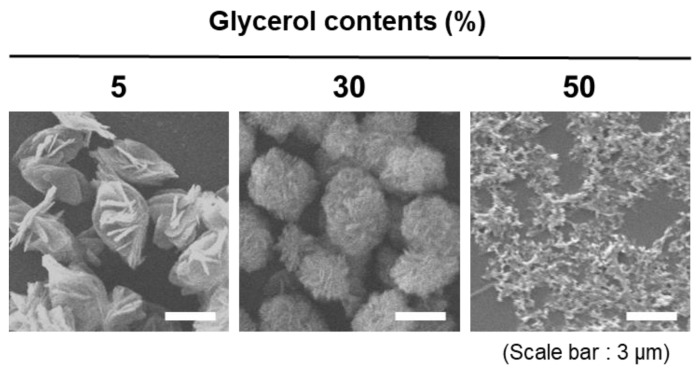
Crystallization trends of magnesium pyrophosphate at different glycerol contents. SEM images of Mg2PPi crystals with different glycerol contents (scale bar: 3 μm).

To further assess the dependence of inorganic crystallization on reaction viscosity, the magnesium pyrophosphate crystals were synthesized without RNA. The tendency to decrease in size was consistent as glycerol contents increased (Figure 3). As the glycerol contents were increased from 5% to 30%, we observed the formation of spherulitic structures with smaller and denser petal structures. In comparison, the apparent crystal structures disappeared at 50% glycerol contents, which proves that the extreme viscosity achieved by 50% glycerol results in inhibited crystalline formation due to a highly limited molecular diffusion. Therefore, we concluded that inhibited crystallization of Mg_2_PPi by viscosity is a factor that affects the RNA microstructure fabrication. Of note, the discrepancy between the exact sizes of RNA microstructures and crystal structures of Mg_2_PPi here comes from the continuous and gradual feeding of the RNA and pyrophosphates in the enzymatic fabrication.

**Figure 4:**
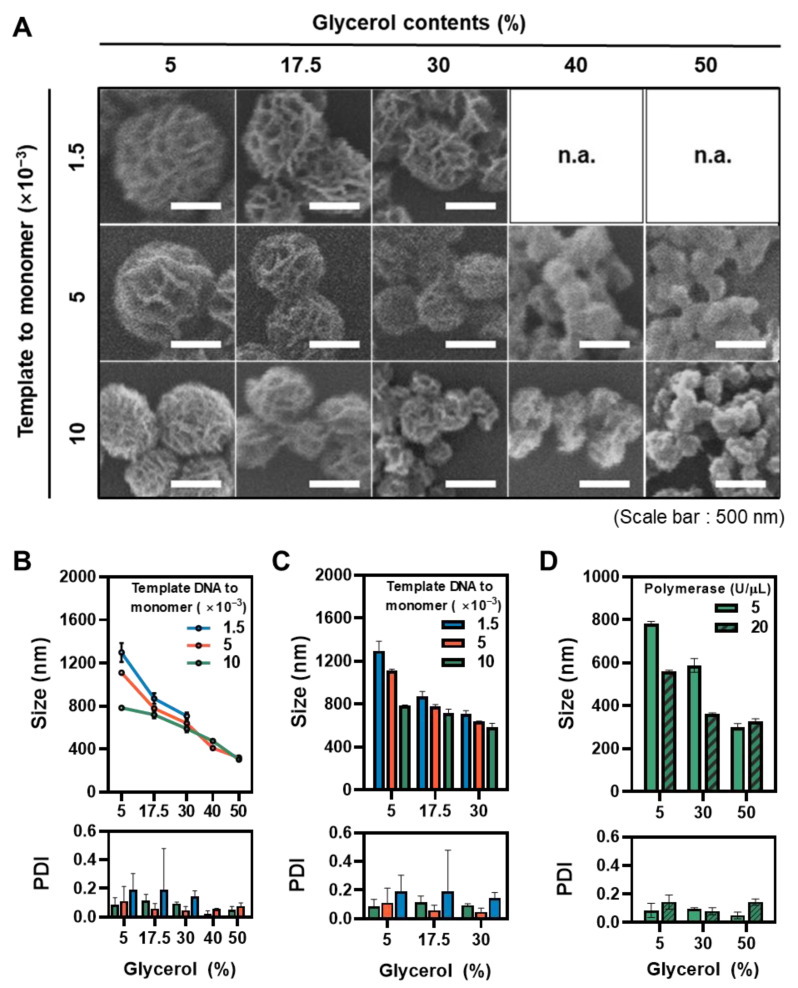
Finely controlled RNA microstructure fabrication through manipulating solution viscosity. (**A**) SEM images of RNA microstructures with different template-DNA-to-monomer ratio (scale bar: 500 nm). Three different reaction conditions were analyzed with the manipulation of glycerol contents. (n.a. = not available data) (**B**) The sizes and PDI of the RNA microstructures at different reaction conditions (n = 3). (**C**) The sizes and PDI of RNA microstructures at a different template to monomer concentrations in the range of 5% to 30% glycerol contents (n = 3). (**D**) The sizes and PDI of the RNA microstructures at a template DNA to monomer = 10 × 10^−3^ condition with different polymerase concentrations (n = 3).

In addition to the previous study, which reported that the increased rNTP concentration is known to reduce the size of RNA particles [7], the effect of manipulating the template-DNA-to-monomer ratio was also evaluated to gain further controllability. By changing the ratio from 10 × 10^−3^ to 1.5 × 10^−3^ at different viscosities, variously sized pools of RNA microstructures were achieved (Figure 4A,B). At all tested template DNA to monomer ratio, the tendency to decrease in size with increased viscosity was preserved (Figure 4C). Of note, over 40% of glycerol contents resulted in minimal differences, which provides further evidence of the solution viscosity becoming the most dominant factor at a highly viscous condition. Similarly, the minimal size changes observed at high glycerol contents with different concentrations of enzymes further strengthen the previous result (Figure 4D). Of note, an increase in the template-DNA-to-polymerase ratio decreased the RNA particle size at all viscosity conditions, which is consistent with previous reports [5,15,16].

Thus far, the enzymatic reaction was controlled rationally here by adjusting a single factor that does not participate in the reaction. At a suboptimal viscosity achieved by 5 to 30% glycerol contents in enzymatic reaction, the increased viscosity resulted in an increased reaction rate with reduced sizes of the final products. In comparison, higher viscosities led to impeded enzyme processing owing to the limited molecular movements. Notably, the rationale was consistent with changes in the active components of the reaction. Furthermore, the various size pools with different RNA contents were also achieved. Although it is not provided in a manuscript, the total costs of 300 nm RNA structure fabrication were reduced by about 25 folds relative to regulation through enzyme concentration.

## 4. Conclusions

In this study, the size of the RNA structure was precisely regulated by a single non-reactive factor, viscosity. The increased viscosity at 30% by the addition of glycerol affected the reaction rate, reducing the sizes of hybrids. On the other hand, the controllability of RNA microstructure fabrication became minimal at higher viscosities owing to the interference in polymerase activity. Furthermore, manipulating reaction components along with viscosity achieves variously sized pools of RNA microstructures, ranging from 300 nm to 1.3 μm. Finally, the required size of the structures is different depending on the biomedical application, and therefore, we suggest new approaches to manipulate the size of RNA microstructures to satisfy diverse biomedical demands precisely and economically. In a follow-up study, the biological response and therapeutic efficiencies of the synthesized RNA microstructures with various sizes should be tested. Moreover, the limitations of noncationic, nucleic acid-based microstructures should be overcome through functionalization of the surface with various engineering tools.

## Figures and Tables

**Table 1:** DNA sequences for synthesizing RNA microstructures. Complementary DNA sequence for promoter region for T7 RNA polymerase is shown in red, and the primer for T7 RNA polymerase binding to the red region to form the promoter region for T7 RNA polymerase.

DNA Strands	Length (nt)	Sequence
Linear DNA 1 (sense)	92	5′—Phosphate—ATA GTG AGT CGT ATT AAA AAC TTC AGG GTC AGC TTG CTT GCT GGA TGA AGG ACG GTC GAA CGC AAA ACT TCA GGG TCA GCT TGC TTA TCC CT—3′
Linear DNA 2 (anti-sense)	92	5′—Phosphate—ATA GTG AGT CGT ATT AAA GCA AGC TGA CCC TGA AGT TTT CTT AGG CTG GAC AAC AAC CAT CTA AAG CAA GCT GAC CCT GAA GTT TTA TCC CT—3′
Primer for T7 RNA polymerase	22	5′—TAA TAC GAC TCA CTA TAG GGA T—3′

## Data Availability

The data presented in this study are available on request from the corresponding author.
